# A Case of Rupture of the Ilium and Omentum, and Fatal Result in Twelve Hours

**Published:** 1850-02

**Authors:** F. H. Gordon

**Affiliations:** Lebanon, Tenn.


Art. II.—*A Case of Rupture of the Ilium and Omentum, and fatal result in twelve hours.*

The following case was reported to me, by my pupil, Henry B. Vaughan.

On the twelfth of the present month, December, Daniel Page, a free man of color, formerly of Cincinnati, Ohio, was riding a mule with speed, and being thrown to the ground, he fell upon the end of his walking cane.—The cane reached the ground end foremost, and standing perpendicularly, the upper end was received by the abdomen, to the right of, and a little below the umbilicus.—About 2 o'clock, P. M., Dr. Crittenden saw him, just after the accident. At 4, P. M., Mr. Vaughan saw him.—He was in great agony. Pain in the epigastrium ; almost constant vomiting ; pulse small, corded and frequent; tenderness, on pressure, over the whole abdomen ; considerable thirst ; constipation of bowels, so obstinate that all efforts to evacuate them per anum were fruitless ; a small hernial tumor was protruded through the right external ring, which was readily reduced by the taxis.

The surface became rapidly cold, and the patient died at 2, A. M., on the 13th.

*Autopsy, by the above two gentlemen, ten hours after death.*—The great omentum was pushed upward and backward, carrying the transverse arch of the colon with it, so as to be completely concealed by the stomach. It was highly injected throughout, and lacerated at several points. Eight or ten ounces of bloody serum in the peritoneal cavity. The peritoneal coat of the intestines, especially of the small bowels, was of a bright red color, and their convolutions were so completely agglutinated everywhere with coagulated lymph, as to form them into one mass. The membrane on the walls of the abdomen, was generally inflamed, and covered in patches with false membrane. There was a transverse rent of the ilum, about six inches above the ileo-cecal valve, extending two thirds of the circumference of the bowel, through which some feces had escaped.

The point of interest in this case, is the extensive adhesions formed in so short a time. The peristaltic action must have been completely paralyzed, so as to allow the lymph to be organized as fast as secreted.

F. H. GORDON.

Lebanon, Tenn., Dec. 15, 1849.

